# Differentiation of Heart Failure Patients by the Ratio of the Scaling Exponents of Cardiac Interbeat Intervals

**DOI:** 10.3389/fphys.2019.00570

**Published:** 2019-05-14

**Authors:** Mirjana M. Platiša, Nikola N. Radovanović, Aleksandar Kalauzi, Goran Milašinović, Siniša U. Pavlović

**Affiliations:** ^1^Institute of Biophysics, Faculty of Medicine, University of Belgrade, Belgrade, Serbia; ^2^Pacemaker Center, Clinical Center of Serbia, Belgrade, Serbia; ^3^Department for Life Sciences, Institute for Multidisciplinary Research, University of Belgrade, Belgrade, Serbia; ^4^Faculty of Medicine, University of Belgrade, Belgrade, Serbia

**Keywords:** heart failure, scaling exponents, detrended fluctuation analysis, Poincaré plot, autonomic cardiac control, asymmetry

## Abstract

Heart failure (HF) is one of the most frequent heart diseases. It is usually characterized with structural and functional cardiac abnormalities followed by dysfunction of autonomic cardiac control. Current methods of heartbeat interval analysis are not capable to differentiate HF patients and some new differentiation of HF patients could be useful in the determination of the direction of their treatment. In this study, we examined potential of the ratio of the short-term and long-term scaling exponents (*α*_1_ and *α*_2_) to separate HF patients with similar level of reduced cardiac autonomic nervous system control and with no significant difference in age, left ventricular ejection fraction (LVEF) and NYHA class. Thirty-five healthy control subjects and 46 HF patients underwent 20 min of continuous supine resting ECG recording. The interbeat interval time series were analyzed using standardized power spectrum analysis, detrended fluctuation analysis method and standard Poincaré plot (PP) analysis with measures of asymmetry of the PP. Compared with healthy control group, in HF patients linear measures of autonomic cardiac control were statistically significantly reduced (*p* < 0.05), heart rate asymmetry was preserved (*C*_up_ > *C*_down_, *p* < 0.01), and long-term scaling exponent *α*_2_ was significantly higher. Cluster analysis of the ratio of short- and long-term scaling exponents showed capability of this parameter to separate four clusters of HF patients. Clusters were determined by interplay of presence of short-term and long-term correlations in interbeat intervals. Complementary measure, commonly accepted ratio of the PP descriptors, SD2/SD1, showed tendency toward statistical significance to separate HF patients in obtained clusters. Also, heart rate asymmetry was preserved only in two clusters. Finally, a multiple regression analysis showed that the ratio *α*_1_/*α*_2_ could be used as an integrated measure of cardiac dynamic with complex physiological background which, besides spectral components as measures of autonomic cardiac control, also involves breathing frequency and mechanical cardiac parameter, left ventricular ejection fraction.

## Introduction

Real-life experience warns us that patients do not have the same clinical response to a single treatment regimen and that traditionally accepted clinical parameters are not good enough predictors of the success of the performed therapy. This is also the case in various areas of the heart failure (HF) treatment. For instance, despite the availability of advanced imaging techniques and strict clinical, echocardiographic and electrocardiographic criteria for the selection of patients with HF and an indication for cardiac resynchronization therapy device implantation, still 20–40% of these patients do not have positive response to this therapy (Dhesi et al., 2017). Therefore, in this field it is necessary to find new ways of separating HF patients, with the intent of defining a group of them who would benefit most from the cardiac resynchronization therapy.

Both intrinsic heart rate and its modulation are primarily determined by alterations in the autonomic tone. The autonomic tone is the general activity rate of the autonomic nervous system (ANS) and it is considered to refer to its long-term activity. It is accepted that measurements of linear time domain and frequency domain variables of heart rate variability (HRV) are simple and practical tools to assess autonomic function (Akselrod et al., 1981; Task Force of the ESC and the NASPE, 1996). However, introduction of methods derived from non-linear dynamics for analyzing HRV have shown new complementary insights into dynamic and structural nature of HRV signals (Eke et al., 2002, 2012; Sassi et al., 2015; Platiša et al., 2016). Since multiple regulatory mechanisms of cardiac control operate across different time scales and have shown scaling behavior, classical linear signal analysis methods are often unsuitable to quantify complex HRV content which is far from simple periodicity. One of the featured groups of non-linear measures in quantifying complex physiological dynamics is a group of fractal measures which are used to assess self-affinity of heartbeat fluctuations over multiple time scales. Fractal organization of healthy sinus rhythm dynamics represents complex output from linear and non-linear processes, usually with non-stationary properties. Long-term scaling properties of interbeat time series was first quantified by the scaling exponent (*β*) as a slope of the regression line of the power versus frequency relation on log-log graph (Kobayashi and Musha, 1982; Saul et al., 1987). In general, the power law scaling exponent is typically calculated in the frequency domain as the *β* or in the time domain as the Hurst exponent (*H*) (Bassingthwaighte et al., 1994; Eke et al., 2000). The technique of detrended fluctuation analysis (DFA), based on modified root mean square analysis of a random walk, was proposed to assess the intrinsic correlation properties of a complex cardiac system where scaling exponent (*α*) of approximately 1 indicates fractal-like behavior of healthy heart rate dynamics (Peng et al., 1995). The obtained exponent is similar to the Hurst exponent, except that DFA may also be applied to non-stationary signals. With this method, the presence of correlations in the fluctuations of heartbeat intervals can be quantified by short- and long-term scaling exponents (*α*_1_ and *α*_2_) in two distinct linear regions that determine range of the short- and long-term correlation properties.

Beside monofractal complexity, multifractal analysis introduced by Ivanov et al. (1999) revealed new informations about physiological complexity of HRV signals. Multifractal complexity arises from a large number of local scaling exponents which physiological background was explained with involvement of coupled cascades of feedback loops in healthy cardiac system. More, Amaral et al. (2001) showed significant impact of parasympathetic control on the multifractal properties on HRV where atropine administration resulted with a marked loss of multifractality. Reduced multifractality also was found in pathological state, in patients with congestive HF (Ivanov et al., 1999). Further, Silva et al. (2014) found that loss of multifractality may indicate an impairment of the left ventricular ejection fraction (LVEF) in patients after acute myocardial infarction. One of the later papers of Ivanov et al. (2009) showed that physiological or physical systems with similar 1/*f* scaling behavior can differ in various levels of complexity which depend on the nature of control mechanisms. The necessity for developing new methods to detect the network between individual organ systems as well as between network of coupled control mechanisms, in response to changes in (patho)physiological conditions, has been recognized in the proposed interdisciplinary area known as a Network Physiology (Lin et al., 2016; Ivanov et al., 2017). This new field focuses on understanding physiological functions with new theoretical framework and analytic formalism.

Fractal view of physiology has become the basis in understanding and controlling physiological networks where both homeostatic and allometric control mechanisms existed (West, 2009). Homeostatic control has a negative feedback character which is local and rapid while allometric control can take into account long-range interactions in complex phenomena (West, 2010). Hence, in recent modeling the dynamics and control of complex physiological phenomena the fractional calculus is more frequently applied (Bogdan et al., 2013). Furthermore, there have been efforts of reconstructing the network between physiological processes when some physiological signals are not observed yet capturing their fractional behavior through fractional order derivatives (Gupta et al., 2018).

In this study, we analyzed heterogeneity of HF patients through application of various methods of HRV analysis. More, we examined potential of the ratio of short- and long-term scaling exponents to differentiate subgroups of HF patients with different short-term and long-term correlation properties. We also calculated standard linear HRV measures with twofold aim. The first one was to show that our data are in line with previously reported results (their reduction and alterations in HF patients) and the second one was to help us to reveal the physiological background of the obtained results.

## Materials and Methods

### Subjects

The group of HF patients comprised 46 patients (nine females) with a mean age of 59 ± 2 years (range 37–78 years). All patients had HF with reduced LVEF, on average below 30%. They all had sinus rhythm without any supraventricular or ventricular rhythm disorders, including supraventricular and ventricular extrasystoles. Most of them were in functional class NYHA II (36 patients) and 10 patients had worse functional capacity (NYHA III). Majority of HF patients in this study were receiving optimal therapy for HF, including β-blockers, angiotensin-converting enzyme (ACE) inhibitors, and aldosterone blockers. An individual therapeutic approach had always been applied to the patients, which means that patients were not exclusively receiving in the guidelines recommended, but maximum tolerated doses of drugs, and rarely, in the presence of certain contraindications, some of previously mentioned group of drugs was left out of therapy. Control healthy group was formed from 35 volunteers (17 females) with a mean age 44 ± 2 years (range 35–59 years). All subjects were non-smokers, without medical history. Ethic Committee of the Faculty of Medicine, Belgrade University approved this study (Ref. Numb. 29/III-5).

### Experimental Protocol and Data Acquisition

Experiments were performed in the morning between 7:00 and 10:00 a.m. in a quiet setting at the Pacemaker Center of the Clinical Center of Serbia and at the Laboratory for Biosignals, Institute of Biophysics, Faculty of Medicine, Belgrade University. Twenty minutes electrocardiogram (ECG) and respiratory signal data were recorded with sampling rate 1 kHz using Biopac and AcqKnowledge 3.9.1 software (BIOPAC System, Inc., Santa Barbara, CA, United States). The ECG data were collected using the ECG 100C electrocardiogram amplifier module. The classic 1 channel ECG for the measurement of Lead I based on three electrodes placed on left and right shoulder and the right leg was used. The RSP 100C respiratory pneumogram amplifier module with TSD 201 transducer attached to the belt (adjustable nylon strap) was used to measure abdominal expansion and contraction. Transducer was placed on the abdomen, at the point of minimum circumference (maximum expiration). Subjects were relaxed before measurement, and they were supine and breathed with spontaneous breathing frequency. Interbeat (RR) intervals and interbreath intervals were extracted from recorded signals using the Pick Peaks tool from Origin 6.0 (Microcal Origin, Northampton, MA, United States). Breathing frequency (BF) was obtained as a reciprocal value of the mean interbreath interval.

### HRV Analysis in Time Domain

A few standard HRV parameters in time domain were determined from time series of RR intervals with our Matlab program: (1) standard deviation of the RR intervals (SDNN), (2) root mean square difference between successive RR intervals, and (3) the percent of RR intervals which were longer by more than 50 ms than the immediately following RR interval (pNN50) (Task Force of the ESC and the NASPE, 1996).

### Frequency Domain Analysis

Heart rate variability analysis in frequency domain was performed using Origin 6.0 (Microcal Origin, Northampton, MA, United States) (Platiša and Gal, 2006; Kapidžić et al., 2014). RR interval series were resampled using the mean RR-value for each subject. Power spectrum densities were obtained using FFT with Hanning window (Microcal Origin, Northampton, MA, United States). Absolute values of spectral components were determined carrying out an integration of the power spectrum in the range of total power (TP, 0.0033–0.4 Hz), very low frequency (VLF, 0.0033–0.04 Hz), low frequency (LF, 0.04–0.15 Hz) and high frequency (HF, 0.15–0.4 Hz) (Task Force of the ESC and the NASPE, 1996). The power of RR variability in VLF range is related to long-term regulation mechanisms related to the thermoregulation, to the renin-angiotensin system and to other humoral factors (Task Force of the ESC and the NASPE, 1996). The physiological interpretation of LF spectral component is still controversial because both sympathetic and parasympathetic contributions are involved in this measure (Task Force of the ESC and the NASPE, 1996; Billman, 2011), while HF spectral power is generally accepted as a marker of parasympathetic activation (Akselrod et al., 1981; Billman, 2011). In order to achieve a normal distribution of the data, natural logarithm of spectral powers in absolute units was taken. Relative units of spectral powers were calculated by dividing each spectral component with the sum of all three spectral powers.

### Detrended Fluctuation Analysis (DFA)

A modification of the random walk model analysis has been used to quantify the fractal-like scaling properties of RR interval time series (Peng et al., 1995). The root-mean-square fluctuations of the integrated and linear detrended data *F*(*n*) were measured in observation windows of varying sizes *n* and then plotted against the size of window on a log–log scale. The power-law behavior was quantified as the slope of the linear regression line, log *F*(*n*) ∼*α* log *n*. This slope is defined as the scaling exponent *α*. In this study detrended fluctuation function *F*(*n*) was calculated by using algorithm from PhysioNet^1^ (Goldberger et al., 2000). The short-term scaling exponent *α*_1_ was calculated over the window size *n* = (4–16) and the long-term scaling exponent *α*_2_ was calculated over the window size *n* ≥ 16. Scaling exponents were estimated with standard errors and the coefficient of determination (*R*^2^) was calculated in OriginPro 8 (OriginLab Corporation, Northampton, MA, United States). An *α* < 0.5 characterizes signal with anticorrelations (with stronger anticorrelations when *α* is closer to 0). If *α* = 0.5 there are no correlations and signal represents (Gaussian) white noise; if *α* ≈ 1 represents 1/*f* noise and if *α* = 1.5 the signal is random walk (Brownian motion) (Peng et al., 1995; Bashan et al., 2008).

### Asymmetry of the Poincaré Plot

A typical HRV Poincaré plot (PP) represents scatter graph of *RR_i+1_* = *f* (*RR_i_*). The two standard parameters SD1 and SD2, called the PP descriptors, describe distribution of points around two diagonals. It is accepted that SD1 describes instant heartbeat intervals variability and quantifies short-term HRV, while SD2 quantifies long-term HRV. Pearson correlation coefficient of PP, noted as *r*, measured the association between all pairs (*RR_i_*, *RR_i_*_+1_) in the time series of RR intervals of one subject. Beside excellent visualization capability and short- and long-term HRV information quantification, the standard PP technique revealed asymmetry as one of unexploited physiological phenomena in resting healthy people (Piskorski and Guzik, 2007). Guzik et al. (2006) extended standard descriptor SD1 (dispersion of the PP across the line of identity, *y* = *x*) to two new finer descriptors SD1*_up_* and SD1*_down_*. Shortly, pattern of heart rate during acceleration is different to the pattern of deceleration, i.e., in deceleration contribution of the points above the line of identity (*RR_i_*< *RR_i_*_+1_) is higher than that of the points below the line (*RR_i_* > *RR_i_*_+1_). They introduced two variables *C_up_* and *C_down_*:

(1) $$C_{\text{up}}=\frac{SD1_{\text{up}}^{2}}{SD1^{2}}$$

(2) $$C_{\text{down}}=\frac{SD1_{\text{down}}^{2}}{SD1^{2}}$$

which determine the relative contribution of *SD*1*_up_* and *SD*1*_down_* to SD1. Analysis of the PP of RR intervals was done with our Matlab program (The MathWorks Inc., United States). Relation between *C_up_* (*C_down_*) and Bauer’s deceleration (acceleration) capacity (Bauer et al., 2006) is explained in the Supplementary Material.

### Statistics

Normal distribution of data was examined by the Shapiro–Wilk test (appropriate for a small sample size of up to 50 subjects). We used natural logarithm of the spectral powers to obtain their normalized values. The K-means cluster analysis with Euclidean distance measure was performed for continuous variable – the ratio of the scaling exponents *α*_1_/*α*_2_. One-way ANOVA was applied to find significant difference in mean values of each variable or parameter: (1) between control and HF group; (2) between four clusters in HF group with Bonferroni *post hoc* test; and (3) between control and each cluster of HF with Bonferroni *post hoc* test. Multiple regression analysis was applied to find which variables and parameters predict the ratio of the scaling exponents in HF patients. Statistical analyses were performed using IBM Statistical Package for the Social Sciences (SPSS) version 19.0. Data are presented as mean ± standard errors. *P* < 0.05 was used as statistically significant.

## Results

Table 1 shows clinically relevant data of HF subjects and descriptive statistic results for all linear and non-linear HRV measures in healthy control group and HF patients. It can be observed that, as it was expected, patients with HF had statistically significantly reduced values of linear measures of autonomic cardiac control compared with healthy control group. However, some quantifiers of HRV properties were not statistically different between groups (*C_up_* and *C_down_*, as well as short-term scaling exponent *α*_1_). Also, younger control subjects had higher heart rate than HF patients (*p* < 0.01).

**Table 1:** Anthropometric data, clinical parameters, and linear and non-linear parameters of heart interbeat intervals in healthy control subjects and heart failure patients.

	Control, *N* = 35 (17 F)	Heart failure, *N* = 46 (9 F)	*p*
Age (years)	44 ± 2	59 ± 2	<0.01
NYHA		2.22 ± 0.10	
LVEF (%)		27.52 ± 0.93	
SDNN (ms)	46.3 ± 2.6	34.1 ± 2.4	<0.01
RMSSD (ms)	31.6 ± 2.8	18.4 ± 1.5	<0.01
pNN50 (%)	11.2 ± 2.1	3.02 ± 0.67	<0.01
SD1 (ms)	16.4 ± 1.4	11.8 ± 1.1	0.013
SD2 (ms)	43 ± 2.4	32.0 ± 2.3	<0.01
*r*, Pearson PP	0.735 ± 0.028	0.727 ± 0.035	0.86
SD2/SD1	2.94 ± 0.16	3.42 ± 0.27	0.16
*C_up_*	55.3 ± 1.0^**^	54.27 ± 0.96^**^	0.47
*C_down_*	44.7 ± 1.0	45.72 ± 0.96	0.46
RR (s)	0.873 ± 0.018	0.938 ± 0.023	<0.01
ln[VLF (ms^2^)]	5.68 ± 0.15	5.08 ± 0.19	0.04
ln[LF (ms^2^)]	5.59 ± 0.15	3.97 ± 0.18	<0.01
ln[HF (ms^2^)]	5.27 ± 0.17	3.86 ± 0.19	<0.01
ln[TP (ms^2^)]	6.72 ± 0.14	5.70 ± 0.17	<0.01
BF (Hz)	0.239 ± 0.10	0.258 ± 0.089	0.49
*α*_1_	0.895 ± 0.029	1.000 ± 0.035	0.22
*α*_2_	0.830 ± 0.022	0.932 ± 0.020	<0.01
*α*_1_/*α*_2_	1.111 ± 0.052	1.099 ± 0.047	0.44

*^∗∗^p < 0.01 (C_up_ vs. C_down_). Data are presented as mean values ± standard errors. LVEF, left ventricular ejection fraction; SDNN, standard deviation of RR intervals; RMSSD, root mean square of successive differences of RR intervals; pNN50, percentage of consecutive RR intervals that deviate from one another by more than 50 ms. SD1, SD2, and r (Pearson PP), C_up_, and C_down_ are parameters of Poincaré plot. RR, mean interbeat interval; VLF, very low frequency spectral component; LF, low frequency spectral component; HF, high frequency spectra component; TP, total power of spectral power density; BF, breathing frequency; α_1_, short-term scaling exponent; α_2_, long-term scaling exponent.*

**FIGURE 1:**
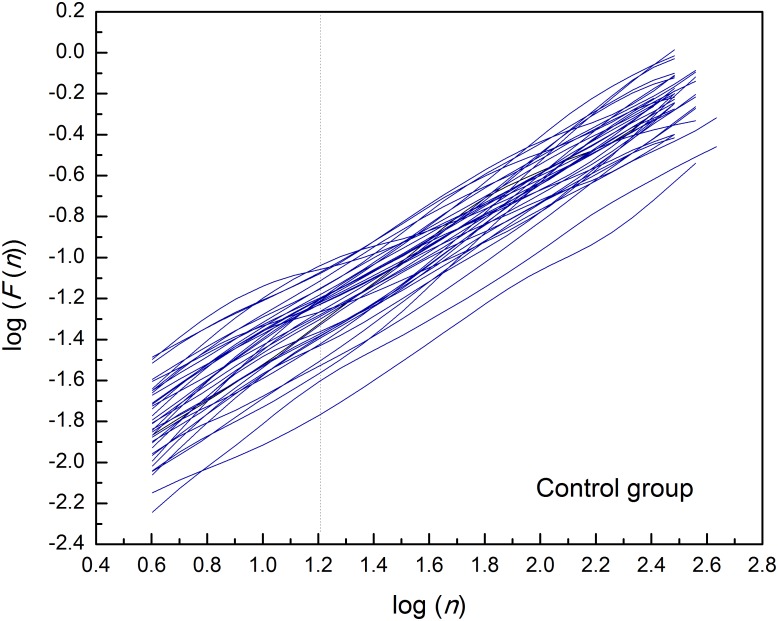
Detrended fluctuation functions *F*(*n*) for time series of interbeat intervals (approximately *N* = 1,200 samples) versus segment size *n* on the log-log graph, in the control group of healthy subjects. Dashed line separates the two ranges in which the scaling exponents have been determined (*n* = 16).

**FIGURE 2:**
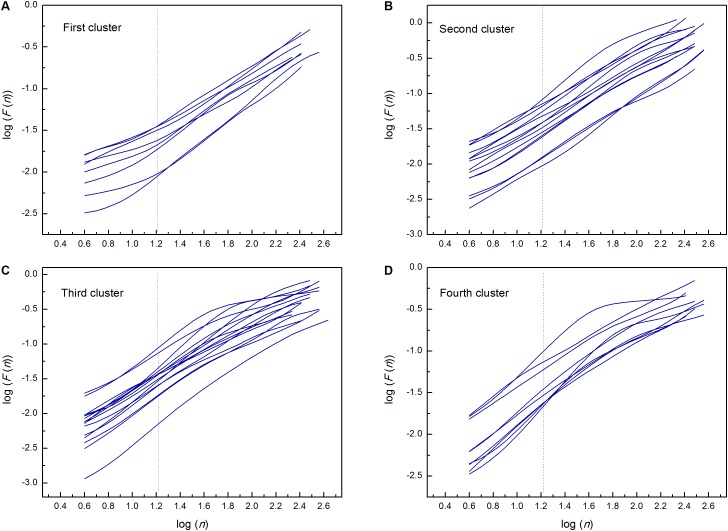
Detrended fluctuation functions *F*(*n*) for time series of interbeat intervals versus segment size *n* on the log-log graph; in the first **(A)**, second **(B)**, third **(C)**, and fourth **(D)** cluster of heart failure patients. Dashed lines separate the two ranges in which the scaling exponents have been determined (*n* = 16). Estimated scaling exponents with the coefficient of determination for every patient in each cluster are given in Tables 2–5.

By applied cluster analysis we found that the ratio of the short- and the long-term scaling exponents, *α*_1_/*α*_2_, was significantly capable to differentiate four clusters of HF subjects. All curves of detrended fluctuation functions *F*(*n*) plotted versus segment size *n* on the log-log graph are presented on Figure 1 for healthy subjects and on Figure 2 for the four clusters of HF patients. Values of estimated scaling exponents for each patient in each cluster of HF group could be found in the Tables 2–5.

**Table 2:** Estimated values of short-term (*α*_1_) and long-term (*α*_2_) scaling exponents in the first cluster of heart failure patients.

Patient	*α*_1_	*R*^2^	*α*_2_	*R*^2^
1	0.728 ± 0.060	0.95	1.143 ± 0.012	0.99
2	0.661 ± 0.011	0.99	1.001 ± 0.014	0.99
3	0.661 ± 0.038	0.98	0.937 ± 0.021	0.99
4	0.536 ± 0.027	0.98	0.8971 ± 0.0037	0.99
5	0.420 ± 0.028	0.97	1.0636 ± 0.0077	0.99
6	0.550 ± 0.012	0.99	0.831 ± 0.051	0.99
7	0.414 ± 0.013	0.99	0.863 ± 0.012	0.99
8	0.512 ± 0.017	0.99	0.940 ± 0.016	0.99

*Values are mean ± standard error. R^2^, coefficient of determination (the goodness of linear fit).*

**Table 3:** Estimated values of short-term (*α*_1_) and long-term (*α*_2_) scaling exponents in the second cluster of heart failure patients.

Patient	*α*_1_	*R*^2^	*α*_2_	*R*^2^
1	1.015 ± 0.041	0.99	1.035 ± 0.027	0.99
2	0.956 ± 0.016	0.99	0.904 ± 0.018	0.99
3	0.948 ± 0.018	0.99	0.954 ± 0.015	0.99
4	0.830 ± 0.022	0.99	1.0147 ± 0.0069	0.99
5	0.964 ± 0.028	0.98	1.1064 ± 0.0094	0.99
6	0.827 ± 0.036	0.99	0.999 ± 0.012	0.99
7	0.898 ± 0.044	0.99	0.929 ± 0.015	0.99
8	1.060 ± 0.043	0.99	1.0073 ± 0.050	0.97
9	0.967 ± 0.033	0.99	1.033 ± 0.038	0.98
10	0.963 ± 0.016	0.99	0.929 ± 0.032	0.99
11	0.992 ± 0.014	0.99	0.9549 ± 0.0059	0.99
12	0.901 ± 0.0.37	0.99	1.021 ± 0.044	0.97
13	0.862 ± 0.044	0.99	0.988 ± 0.020	0.99
14	1.0029 ± 0.0088	0.99	1.227 ± 0.012	0.99

*Values are mean ± standard error. R^2^, coefficient of determination.*

**Table 4:** Estimated values of short-term (*α*_1_) and long-term (*α*_2_) scaling exponents in the third cluster of heart failure patients.

Patient	*α*_1_	*R*^2^	*α*_2_	*R*^2^
1	1.301 ± 0.033	0.99	1.065 ± 0.025	0.99
2	1.238 ± 0.016	0.99	0.950 ± 0.018	0.99
3	1.187 ± 0.037	0.99	1.031 ± 0.015	0.99
4	1.119 ± 0.029	0.99	0.853 ± 0.019	0.99
5	0.965 ± 0.050	0.98	0.771 ± 0.038	0.97
6	0.978 ± 0.037	0.99	0.778 ± 0.017	0.99
7	0.959 ± 0.018	0.99	0.8275 ± 0.0086	0.99
8	1.104 ± 0.062	0.98	0.892 ± 0.066	0.92
9	1.137 ± 0.038	0.98	0.557 ± 0.051	0.88
10	1.339 ± 0.018	0.99	1.189 ± 0.040	0.99
11	1.162 ± 0.041	0.99	0.909 ± 0.016	0.99
12	1.088 ± 0.020	0.99	0.865 ± 0.035	0.97
13	0.944 ± 0.032	0.99	0.697 ± 0.022	0.98
14	1.017 ± 0.016	0.99	0.841 ± 0.029	0.99
15	1.1356 ± 0.0083	0.99	0.968 ± 0.024	0.98

*Values are mean ± standard error. R^2^, coefficient of determination.*

**Table 5:** Estimated values of short-term (*α*_1_) and long-term (*α*_2_) scaling exponents in the fourth cluster of heart failure patients.

Patient	*α*_1_	*R*^2^	*α*_2_	*R*^2^
1	1.060 ± 0.025	0.99	0.7804 ± 0.0081	0.99
2	1.235 ± 0.018	0.99	0.847 ± 0.030	0.99
3	1.179 ± 0.058	0.99	0.828 ± 0.037	0.98
4	0.9603 ± 0.0018	0.99	0.739 ± 0.020	0.99
5	1.3185 ± 0.052	0.99	0.869 ± 0.065	0.91
6	1.1010 ± 0.0046	0.99	0.729 ± 0.029	0.97
7	1.3314 ± 0.0092	0.99	0.8894 ± 0.019	0.99
8	1.412 ± 0.011	0.99	0.504 ± 0.062	0.81
9	1.2142 ± 0.0089	0.99	0.863 ± 0.011	0.99

*Values are mean ± standard error. R^2^, coefficient of determination.*

**FIGURE 3:**
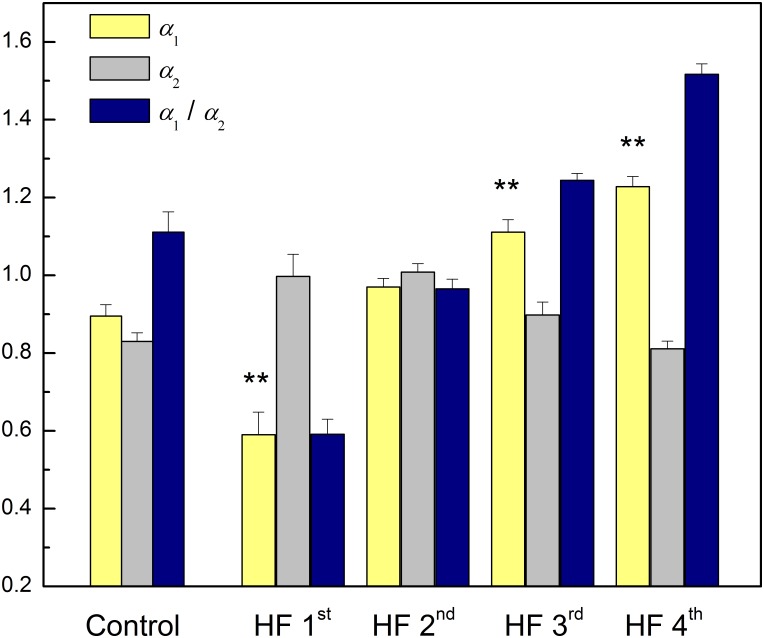
Mean values plus standard errors of short-term scaling exponent (*α*_1_), long-term scaling exponent (*α*_2_) and their ratio *α*_1_/*α*_2_ in control group and four clusters of heart failure patients. ^∗∗^*p* < 0.01 (*α*_1_ vs. *α*_2_). Short-term scaling exponent (*α*_1_) values were significantly different between control group and three clusters (1st, 3rd, and 4th), *p* < 0.01. Long-term scaling exponent (*α*_2_) values were significantly different between control group and two clusters (1st and 2nd), *p* < 0.01. The ratio *α*_1_/*α*_2_ was significantly different between control group and two clusters (1st and 4th), *p* < 0.01, and with tendency toward statistical significance with the 3rd cluster (*p* = 0.07). After comparisons only between clusters for *α*_1_ we obtained following significances: (1st vs. 2nd, 1st vs. 3rd, and 1st vs. 4th, *p* < 0.01) and (2nd vs. 3rd, and 2nd vs. 4th, *p* ≤ 0.01). For *α*_2_ we obtained: (1st vs. 4th, *p* < 0.01) and (2nd vs. 3rd, *p* = 0.06 and for 2nd vs. 4th, *p* < 0.01). For the ratio *α*_1_/*α*_2_ all comparisons were statistically significant (*p* < 0.01).

Table 6 reports results of cluster analysis, with the mean and standard errors of all linear and non-linear HRV indexes for each cluster. Averaged values of short-term scaling exponent (*α*_1_), long-term scaling exponent (*α*_2_), as well as their ratio for each cluster and control group of healthy subjects are shown on Figure 3. It can be seen that in HF patients with reduced autonomic cardiac control, interplay of different correlation properties in heart rhythm over short-term and long-term scales determines four independent subgroups. In comparison of clusters with control group we found that there is no significant difference in *α*_1_ neither in the ratio *α*_1_/*α*_2_ between control and 2nd cluster, while *α*_2_ was significantly lower in control group than in the first two clusters (Figure 3).

**Table 6:** Comparison of clinical data, and linear and non-linear HRV measures between four clusters of heart failure patients.

	First (*N* = 8)	Second (*N* = 14)	Third (*N* = 15)	Fourth (*N* = 9)	*p*
Age (years)	62.1 ± 2.7	55.4 ± 2.5	61.1 ± 2.3	59.2 ± 3.2	0.85
NYHA	2.25 ± 0.16	2.21 ± 0.11	2.27 ± 0.12	2.11 ± 0.11	0.28
LVEF (%)	28.1 ± 2.7	25.8 ± 1.7	28.9 ± 1.7	27.3 ± 1.7	0.61
SDNN (ms)	26,6 ± 4.4	41.8 ± 4.8	31.0 ± 3.5	34.0 ± 6.5	0.15
RMSSD (ms)	20.4 ± 3.0	21.9 ± 3.1	16.4 ± 2.1	14.8 ± 3.5	0.29
pNN50 (%)	2.3 ± 1.2	4.9 ± 1.7	2.19 ± 0.82	2.1 ± 1.1	0.34
SD1 (ms)	14.8 ± 2.6	10.7 ± 1.6	13.0 ± 2.5	8.83 ± 6.4	0.37
SD2 (ms)	25.6 ± 4.2	36.2 ± 4.8	30.9 ± 3.5	33.1 ± 6.4	0.50
*r*, Pearson PP	0.44 ± 0.10	0.818 ± 0.029	0.707 ± 0.061	0.872 ± 0.018	< 0.01
SD2/SD1	2.06 ± 0.48	3.95 ± 0.62	3.25 ± 0.43	4.10 ± 0.33	0.07
RR (s)	1.027 ± 0.043	0.932 ± 0.041	0.910 ± 0.047	0.916 ± 0.044	0.24
ln[VLF (ms^2^)]	4.10 ± 0.43	5.40 ± 0.37	5.16 ± 0.31	5.31 ± 0.30	0.36
ln[LF (ms^2^)]	2.88 ± 0.43	4.04 ± 0.34	3.93 ± 0.26	4.88 ± 0.28	0.06
ln[HF (ms^2^)]	4.12 ± 0.35	4.10 ± 0.34	3.47 ± 0.35	3.89 ± 0.47	0.46
ln[TP (ms^2^)]	5.06 ± 0.35	5.88 ± 0.36	5.66 ± 0.28	6.07 ± 0.31	0.79
rVLF (%)	43.4 ± 7.3	63.5 ± 3.6	63.1 ± 4.7	53.8 ± 6.5	0.045
rLF (%)	12.6 ± 2.4	17.8 ± 2.0	21.9 ± 3.4	32.1 ± 3.2	0.002
rHF (%)	44.0 ± 7.5	18.6 ± 2.2	15.0 ± 3.0	14.1 ± 4.1	0.001
BF (Hz)	0.247 ± 0.022	0.252 ± 0.017	0.265 ± 0.016	0.266 ± 0.020	0.74

*Data are presented as mean values ± standard errors. LVEF, left ventricular ejection fraction; SDNN, standard deviation of RR intervals; RMSSD, root mean square of successive differences of RR intervals; pNN50, percentage of consecutive RR intervals that deviate from one another by more than 50 ms. SD1, SD2, and r (Pearson PP), C_up_, and C_down_ are parameters of Poincaré plot. RR, mean interbeat interval. Absolute and relative values of VLF, very low frequency spectral component; LF, low frequency spectral component; HF, high frequency spectral component; TP, total power of spectral power density; BF, breathing frequency.*

**FIGURE 4:**
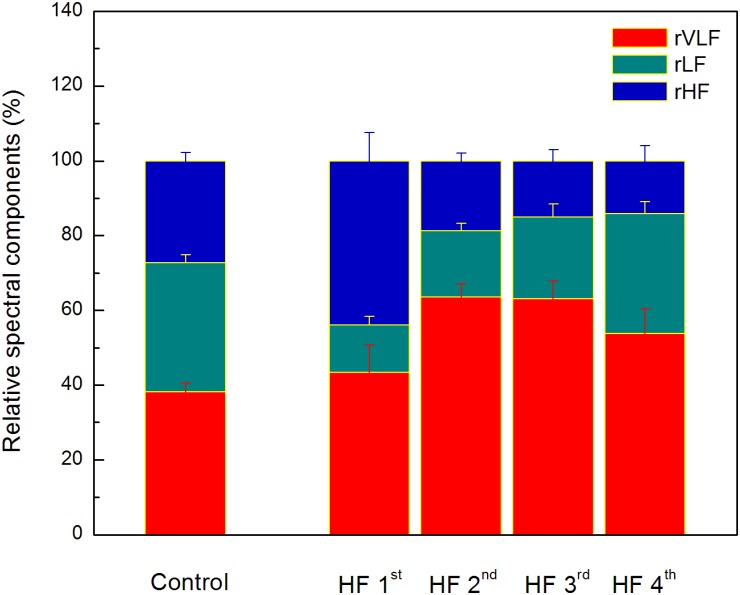
The distribution of relative spectral components in clusters determined by the ratio of the scaling exponents. Values are given as mean plus standard errors. Relative VLF spectral component (rVLF) was significantly different between control group and two clusters (2nd and 3rd), *p* < 0.01. Relative LF spectral component (rLF) was significantly different between control group and three clusters (1st, 2nd, and 3rd), *p* < 0.01. Relative HF spectral component (rHF) was significantly different between control group and two clusters (1st and 3rd), *p* < 0.05, and with tendency toward statistical significance with the 4th cluster (*p* = 0.08). Significant and toward significant differences of comparison between clusters of heart failure patients are for rVLF (1st vs. 2nd, *p* = 0.07 and 1st vs. 3rd, *p* = 0.08), for rLF (1st vs. 4th, *p* < 0.01, and 2nd vs. 4th, *p* = 0.01), and for rHF (1st vs. 2nd, 1st vs. 3rd, and 1st vs. 4th, *p* < 0.01).

Comparison of heart rate dynamics indices between patients with HF showed that there is no significant difference between time HRV measures neither between absolute values of spectral components. However, we found significant difference between subgroups in relative values of spectral powers as well as in their comparison with control group (Figure 4). Results of statistical analysis could be found in the legend of the Figure 4. Also, there is no significant difference with respect to age, NYHA, LVEF, or breathing frequency between clusters patients (Table 6).

**FIGURE 5:**
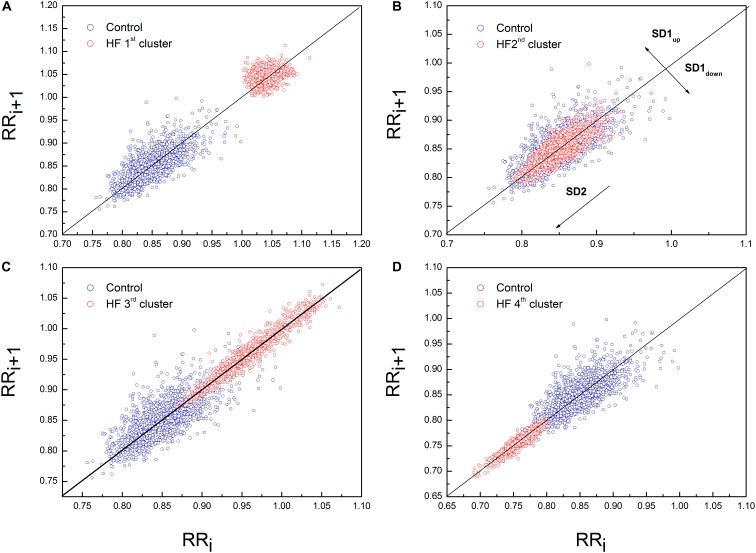
Representative example of Poincaré plot in the first **(A)**, second **(B)**, third **(C)**, and fourth **(D)** cluster of heart failure patients with PP for one healthy subject. Patient from the first cluster had SD1 = 10.10 ms, SD2 = 12.80 ms, *C_up_* = 62.72% and *C_down_* = 37.28%. Patient from the second cluster had SD1 = 7.50 ms, SD2 = 22.30 ms, *C_up_* = 47.42% and *C_down_* = 52.58%. Patient from the third cluster had SD1 = 4.90 ms, SD2 = 35.30 ms, *C_up_* = 53.8% and *C_down_* = 46.2%. Patient from the fourth cluster had SD1 = 3.1 ms, SD2 = 17.7 ms, *C_up_* = 42.63% and *C_down_* = 57.37%. Control subject had SD1 = 13 ms, SD2 = 32 ms, *C_up_* = 57.42% and *C_down_* = 42.76%.

**FIGURE 6:**
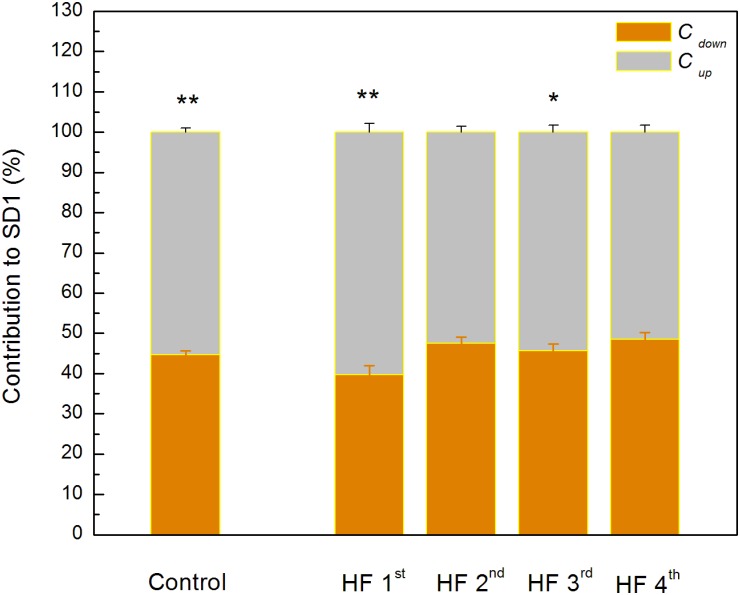
Guzik’s variables of asymmetry in clusters of heart failure patients. Data are presented as mean values plus standard errors. ^∗∗^*p* < 0.01, ^∗^*p* < 0.05 (*C_up_* vs. *C_down_*). There is no significant difference between control group and each of clusters with heart failure patients.

Poincaré plot descriptors SD1 and SD2 showed that they were not able to separate patients in obtained clusters, but there was a tendency toward statistical significance for their ratio SD2/SD1 (Table 6 and Figure 5). Asymmetry variables *C_up_* and *C_down_*, determined from the Poincaré plots, indicated that HF patients could be separated only into two subgroups: one with dominant deceleration mechanism and the second one, where there was no statistical difference between deceleration and acceleration pattern of regulatory mechanisms (Figure 6). We also found that there is no statistical difference between control group and each cluster in *C_up_* and *C_down_*.

A multiple regression analysis was performed to determine predictors of the ratio *α*_1_/*α*_2_. We found that relative spectral powers (rHF and rVLF), the LVEF, normalized total spectral power, and breathing frequency statistically significantly predicted *α*_1_/*α*_2_ in HF subjects, *F*(5,40) = 20.966, *p* < 0.01, *R*^2^= 0.724 (Table 7). This result indicates complex physiological background of the ratio of scaling exponents which comprised relative cardiac vagal and central autonomic control, mechanical efficiency of the left ventricle, total variability as well as modulatory effect of the breathing process.

**Table 7:** Multiple regression analysis with predictors for the ratio of short-term to long-term scaling exponents.

	*b*	S.E. *b*	*β*	*t*	*p*
Constant	1.493	0.259		5.772	0.000
rHF (%)	-2.518	0.272	-1.318	-9.245	0.000
rVLF (%)	-1.468	0.245	-0.863	-6.004	0.000
LVEF (%)	0.014	0.005	0.273	3.023	0.004
ln[TP (ms^2^)]	0.064	0.024	0.230	2.632	0.012
BF (Hz)	0.932	0.459	0.177	2.031	0.049

*rHF, relative spectral component of high frequency domain; rVLF, relative spectral component of very low frequency domain; LVEF, left ventricular ejection fraction; ln TP, natural logarithm of total power; BF, breathing frequency. b, unstandardized regression coefficient; S.E.b, standard error of b; β, standardized regression coefficient.*

## Discussion

In the last few decades DFA method, i.e., short-term and long-term scaling exponents separately, showed a greater prediction potential in several cardiac diseases than any parameter from HRV analyses. In this study, we found that in HF patients short- and long-term correlation properties quantified by scaling exponents could gradually change in the opposite directions. We introduced new parameter, the ratio of short- and long-term correlation properties of heart interbeat fluctuations, which could be used as an integrative parameter of regulatory cardiac mechanisms in HF patients. This parameter is capable to differentiate four clusters which could not be simply classified by some clinical parameters (LVEF or NYHA) or solely by linear and non-linear measures of autonomic cardiac control.

The finding for the first cluster could be a good example of previously recognized interactions between short-term and long-term cardiovascular control mechanisms under specific pathological conditions (Hoyer et al., 2007). Namely, Hoyer et al. (2007) showed that cardiovascular system incorporates dominant short- and long-term control mechanisms which are in optimal adjustment in normal healthy conditions. Under pathological conditions, realized with increased collapse of the short feedback loop, the inverse association between their randomness has been recognized. As a system inherent type of readjustment, short-term randomness increased and long-term randomness decreased. We found in the first cluster of HF patients that the ratio of the scaling exponents had the lowest value; *α*_1_ was significantly reduced and significantly lower than *α*_2_. The short-term scaling exponent has shown greater clinical discrimination of various cardiac diseases. A reduced *α*_1_ indicates loss of fractal organization in cardiac interbeat intervals and it is a good predictor of mortality in post-infarction patients (Huikuri et al., 2000; Tapanainen et al., 2002), specific risk factor for cardiac death in the elderly and it has been proposed as a strong predictor for HF patients (Ho et al., 1997; Mäkikallio et al., 2001). In this state of cardiac system with reduced ANS activity, loss of short-term correlations in heartbeat intervals could result from reduced capacity for cardiovascular adaptation, vagal tone (high percentage of rHF) and/or alterations in breathing pattern independent on the breathing frequency. This is an interesting finding because there are plenty of data on factors that affect the breathing frequency in HF, such as the effects of some drugs like sedatives, or LVEF, NYHA, some lung diseases, but we have not found out enough about alterations in breathing patterns so far (Forleo et al., 2015). In our earlier papers we showed that fractal organization of interbeat intervals dynamic could also be altered with some physiological processes like standing, exercise, and recovery (Platiša and Gal, 2008). Additionally, in our study with voluntary breathing at different breathing frequencies we found reduction of *α*_1_ with increase in breathing frequency (Platiša and Gal, 2010). Perakakis et al. (2009) also showed that breathing frequencies may bias evaluation of short-term fractal scaling properties.

Compared with control, in patients from this cluster fractal long-term correlations become stronger (*α*_2_ increased). Some previous studies have reported change of fractal scaling behavior with aging, where both scaling exponents increased with aging (Ryan et al., 1994; Lipsitz, 1995; Pikkujämsä et al., 1999). That type of correlations is usually related to the rigidness of regulatory mechanisms with reduced control ability. According Guzik’s variables of asymmetry these patients also preserved asymmetry in short HRV, as it is observed in healthy subjects, which indicates that dominant deceleration mechanism had a different pattern from acceleration mechanism/s (Guzik et al., 2006). Relative spectral powers of very low frequency (rVLF) and of high frequency (rHF) regions had similar percentage values and we assumed that regulatory mechanisms in this range of frequencies are equally involved in the state of reduced autonomic cardiac control as the result of readjustment in system control mechanisms.

In the second cluster we did not find statistically significant crossover between scaling exponents (fractal correlations are very similar in both ranges). This cluster is very similar to the control group, but with significantly higher long-term scaling exponent *α*_2_. However, comparison of distributions of relative spectral powers revealed similar rHF, but with a significant reorganization of rLF and rVLF and with domination of slower regulatory mechanism from domain of very low frequencies in these HF patients. More, asymmetry was not reached, and the pattern of short-term deceleration and acceleration mechanisms was not different. We only may speculate that patients from second cluster were in state with some developed compensatory mechanisms of cardiac control.

Regarding relationship between scaling exponents, the third and fourth cluster were similar, i.e., short-term scaling exponent is significantly higher than long-term. Compared with control group, *α*_1_ gradually increased while *α*_2_ remained statistically unchanged. This type of interactions between short-term and long-term randomness under HF as pathological condition, also may be another type of system inherent readjustment, here in the opposite direction compared with the first cluster. In the literature, it can be found that the increase in *α*_1_ is usually related to vagal withdrawal (Tulppo et al., 2001; Castiglioni et al., 2011) but in this HF states probably with preserved feedback loops of regulatory mechanisms. Tendency to decreased *α*_2_ in the fourth cluster may be related to sinus node dysfunction (Shin et al., 2011) or the β-receptor blockade (Castiglioni et al., 2011). It is believed that complexity of interbeat interval fluctuations is generated solely by ANS activity and that quantified linear and non-linear parameters of HRV are quantifiers of modulatory mechanisms of ANS. However, recently published data revealed that interbeat intervals reflect intrinsic complexity with origin in sinoatrial node cells (Ponard et al., 2007; Yaniv et al., 2013) which usually is integrated in the whole complexity of the heart rhythm. Hence, Yaniv et al. (2013) concluded that HRV is determined by the intrinsic properties of cells in the sinoatrial node and the competing influences of the two branches of the autonomic neural input. With these findings, the importance of assessments of long- term scaling exponent has increased. Hotta et al. (2005) showed that *α*_2_ is a more powerful measure for predicting cardiac morbidity and mortality. In the study of Shin et al. (2011) long-term scaling exponent was the only parameter which was capable of discriminating differences in heart rate dynamics between patients with sinus node dysfunction. They concluded that reduced value of *α*_2_ is a robust measure and could be an adjunctive tool for improvement of diagnostic performance in detection of sinus node dysfunction. The question is whether this finding can be applied to our patients, because they were mostly treated with highly selective beta blockers, whereas in the previously mentioned study, non-selective propranolol was used in blocking the beta receptors. Also, in healthy subjects in a much lesser extent, such alterations of scaling exponents are characterized as age-related degradation of integrated physiological regulatory systems (Iyengar et al., 1996). Our patients probably had superposition of both effects which are more pronounced in the fourth cluster. The reason for decrease of *α*_2_ may be related to some other physiological background (Figure 2D). In the sixth and eighth patient, even with statistically approved linear regression analysis, a new scaling regime which is probably related to some slower regulatory mechanism could be identified. Unfortunately, our time series of 20 min length with approximately 2^10^ points was not long enough to detect this regime in all patients of the fourth cluster.

It can be observed that the third and fourth cluster are different with respect to the distribution of asymmetry variables. While there was asymmetry in the third cluster, in the fourth cluster short-term asymmetry has ceased to exist. In order to clarify all these findings, future studies with longer recordings and/or pharmacological recognition need to be done.

We also noticed that even younger control subjects had higher heart rate than HF patients. If it is known that the main pathophysiological feature of HF is the imbalance of ANS with increased sympathetic activity, then it is expected that patients with HF will have faster resting rate than control subjects (Hori and Okamoto, 2012). However, all patients with HF in this study were receiving β-blockers, and some of them were treated with additional antiarrhythmics, such as amiodarone or digoxin. Thus, in all these patients there was iatrogenic decrease of heart rate. Also, in previous study we found that HF patients with sinus rhythm and without ventricular extrasystoles have a significantly lower heart rate compared to HF patients with arrhythmias, either ventricular or supraventricular (Radovanović et al., 2018). Finally, it should be added that some researchers suggest the existence of selectivity of effects of aging on autonomic function in healthy subjects; more precisely, they believe that sympathetic function remains unchanged with increasing age (Parashar et al., 2016).

Results of multiple regression analysis showed that the ratio of the scaling exponents was significantly predicted with the small alterations in five independent measures which determined four clusters of HF patients. Comparison of standardized regression coefficient *β* values showed that relative spectral components, rHF and rVLF, had strong negative relationship with the ratio of the scaling exponents. Relative strength of relationships of LVEF and lnTP with this ratio was much weaker, while with BF it was the weakest.

Recognized heterogeneity in HF patients points to the necessity of introducing new approaches in the analysis of cardiac dynamics which will comprise the interactions with the other coupled physiological systems (Ivanov et al., 2017; Kuhnhold et al., 2017). In this pathological condition, analyses of the heart–brain interactions are of special importance because of recognition and quantification of neuroplasticity changes in the dynamics of the brain stem integrators.

### Limitations

The significant limitation of this study is a statistically significant age difference between healthy controls and HF patients. It is known that aging, as well as diseases, is accompanied by significant cardiovascular modifications, both structural and functional, although there are studies that indicate that fractal temporal organization of cardiac dynamics does not break down with healthy aging (Ferrari, 2002; Schmitt et al., 2009). What is sure is that with aging the sympathetic activity increases, the renin-angiotensin-aldosterone system activity decreases, respiratory sinus arrhythmia and HRV are reduced, as well as effectiveness of cardiovascular and cardiopulmonary reflexes (Ferrari, 2002; Voss et al., 2015). Some of these changes are also a characteristic of HF and, therefore, a large problem is separating truly HF dependent alterations from those arising from aging. However, we could not age-match our HF patients and healthy controls, because it was not feasible to select subjects without any medical history, even without any risk factor for cardiovascular disease development, among those who were closer in age to the HF patients. In our study, patients were treated with a combination of medications from groups of drugs recommended for the treatment of HF. The differences among patients in doses of given drugs remain an inevitable limitation of this research, since the prescribed dose of the drug was determined by the patient’s comorbidities, which were numerous in our patients.

After this study it is necessary to examine the clinical potential of the ratio of short- and long-term scaling exponents, which was the basis for separating of HF patients. We want to determine whether patients of one of these clusters have greater benefit from the cardiac resynchronization therapy. Results of a multiple regression analysis, more precisely, the fact that the examined ratio is at the center of neural, cardiac and respiratory influences, encourages us that this parameter could contribute to a better selection of candidates for this therapy and also be the parameter on which we will rely on in the course of device programming optimization during follow-up period. In the future, this ratio could potentially be used by a device algorithm that would automatically optimize its function. This will surely be the topic of our next research.

## Conclusion

Our findings showed that in the integral cardiac control quantified by the ratio of short-term and long-term scaling exponents, beside neural cardiac control, mechanical properties of the heart and the modulatory effect of the breathing frequency are significantly involved. This ratio is able of differentiating four clusters of HF patients in sinus rhythm which do not differ in cardiac autonomic control, age, LVEF and NYHA class.

## Ethics Statement

This study was carried out in accordance with the recommendations of the Declaration of Helsinki. The protocol was approved by the Ethic Committee of the Faculty of Medicine, Belgrade University (Ref. Numb. 29/III-5). All subjects gave written informed consent in accordance with the Declaration of Helsinki.

## Author Contributions

MP, NR, and SP conceptualized the study. MP and NR designed the experiments, performed the study, wrote the manuscript, and collected and analyzed the data. AK developed program for calculation Poincaré plot descriptors and variables of asymmetry. GM and SP conducted clinical examination and recruitment of patients. All authors discussed, edited, and approved the final and revised version of the manuscript.

## Conflict of Interest Statement

The authors declare that the research was conducted in the absence of any commercial or financial relationships that could be construed as a potential conflict of interest.

## Footnotes

1 https://physionet.org/physiotools/dfa/
