# Supplementary material for: Differentiation of Heart Failure Patients by the Ratio of the Scaling Exponents of Cardiac Interbeat Intervals
Source: Front Physiol. 2019 May 14;10:570. doi: 10.3389/fphys.2019.00570 (PMC6527786; doi:10.3389/fphys.2019.00570)
Supplement: Supplementary file 1 [file Data_Sheet_1.doc]

**Relation between Cup (Cdown) and Bauer's deceleration (acceleration) capacity**

As stated in Guzik et al. (2006), the quantity SD1^2^ is defined as the sum of squared distances of points in Poincaré plot (PP) from the identity line (y=x), divided by the number of points, *n*:

$SD1^{2}=\frac{1}{n}\sum_{i=1}^{n} D_{i}^{2}$. (1)

Heart rate deceleration is related to those points which are positioned above the identity line, where RR_i+1_>RR_i_. Opposite is valid for acceleration. Thus, eq. (1) can further be expressed as the sum of these two subgroups of distances:

$SD1^{2}=SD1_{up}^{2}+SD1_{down}^{2}=\frac{1}{n}\sum_{i=1}^{nup} u_{i}^{2}+\frac{1}{n}\sum_{k=1}^{ndown} d_{k}^{2}$.

It is not difficult to derive analytical expressions for these two components

$SD1^{2}=SD1_{up}^{2}+SD1_{down}^{2}=\frac{1}{n}\sum_{i=1}^{nup} \frac{(RR_{i+1}-RR_{i})^{2}}{2}_{(RR_{i+1}>RR_{i})}+\frac{1}{n}\sum_{k=1}^{ndown} \frac{(RR_{i}-RR_{i+1})^{2}}{2}_{(RR_{i}>RR_{i+1})}$.

Finally, one can obtain their normalized versions *C_up_* and *C_down_* :

$$C_{up}=\frac{SD1_{up}^{2}}{SD1^{2}};C_{down}=\frac{SD1_{down}^{2}}{SD1^{2}}$$

In terms of quantities defined in Bauer et al. (2006), the points positioned above the identity line correspond to deceleration anchors. Thus, the averaged RR interval longer than its preceding RR interval, denoted as X(0), would correspond to averaging all *RR_i+1_* points above the identity line

$X(0)=\frac{1}{nup}\sum_{i=1}^{nup} RR_{i+1}$, *RR_i+1_*>*RR_i_*.

Similarly, average of all first preceding intervals, X(-1), could be expressed as

$X(-1)=\frac{1}{nup}\sum_{i=1}^{nup} RR_{i}$, *RR_i+1_*>*RR_i_*.

An example of how (X(-1),X(0)) point is positioned on a typical PP plot is given on Fig. 1, marked with a red circle.

Fig. 1. Position of elements of Deceleration Capacity, X(0) and X(-1), on a typical Poincaré plot. The red dot is obtained by averaging points which lay above the identity line (thick black).

Deceleration capacity incorporates difference of these two quantities, X(0)-X(-1), as part of the *DC* (deceleration capacity). Let us mark this part of *DC* as *DC_p_*:

$SD1_{up}^{2}=\frac{1}{n}\sum_{i=1}^{nup} \frac{(RR_{i+1}-RR_{i})^{2}}{2}_{(RR_{i+1}>RR_{i})}$(2) $DCp=(X(0)-X(-1))/2=\frac{1}{nup}\sum_{i=1}^{nup} \frac{(RR_{i+1}-RR_{i})}{2}_{(RR_{i+1}>RR_{i})}$(3).

They differ in two main points:

1) in *SD1^2^_up_* (*C_up_)* differences are squared, while in *DCp* they are linear, although all summands are positive;

2) the whole definition of *DC* involves averaging, after phase rectification, of other two adjacent points, X(1) and X(-2), while in *C_up_* only the first neighboring pair is taken into account.

Similarity of these two measures follows from the fact that there is a direct correlation between *SD1^2^_up_* (hence also *C_up_*) and *DCp*, as is evident from eqs. (2) and (3). Geometrical argument is that the red point in Figure 1 is moving away from the identity line if both X(0)-X(-1) or *C_up_* increase.
